# Genomic regions associated with important seed quality traits in food-grade soybeans

**DOI:** 10.1186/s12870-020-02681-0

**Published:** 2020-10-23

**Authors:** Rachel M. Whiting, Sepideh Torabi, Lewis Lukens, Milad Eskandari

**Affiliations:** Department of Plant Agriculture, University of Guelph, Guelph, ON Canada

**Keywords:** Food-grade soybean, Protein, Sucrose, Seed weight, Linkage analysis, Candidate genes

## Abstract

**Background:**

The production of soy-based food products requires specific physical and chemical characteristics of the soybean seed. Identification of quantitative trait loci (QTL) associated with value-added traits, such as seed weight, seed protein and sucrose concentration, could accelerate the development of competitive high-protein soybean cultivars for the food-grade market through marker-assisted selection (MAS). The objectives of this study were to identify and validate QTL associated with these value-added traits in two high-protein recombinant inbred line (RIL) populations.

**Results:**

The RIL populations were derived from the high-protein cultivar ‘AC X790P’ (49% protein, dry weight basis), and two high-yielding commercial cultivars, ‘S18-R6’ (41% protein) and ‘S23-T5’ (42% protein). Fourteen large-effect QTL (R^2^ > 10%) were identified associated with seed protein concentration. Of these QTL, seven QTL were detected in both populations, and eight of them were co-localized with QTL associated with either seed sucrose concentration or seed weight. None of the protein-related QTL was found to be associated with seed yield in either population. Sixteen candidate genes with putative roles in protein metabolism were identified within seven of these protein-related regions: qPro_Gm02–3, qPro_Gm04–4, qPro_Gm06–1, qPro_Gm06–3, qPro_Gm06–6, qPro_Gm13–4 and qPro-Gm15–3.

**Conclusion:**

The use of RIL populations derived from high-protein parents created an opportunity to identify four novel QTL that may have been masked by large-effect QTL segregating in populations developed from diverse parental cultivars. In total, we have identified nine protein QTL that were detected either in both populations in the current study or reported in other studies. These QTL may be useful in the curated selection of new soybean cultivars for optimized soy-based food products.

## Background

Soybean [*Glycine max* (L.) Merrill] is a major source of plant-based dietary protein. An increased demand for whole-bean soy-based food products, such as tofu and soymilk, in western countries has attracted the attention of researchers, soybean growers and soy-based food processors. Soy-based products require specific physical and chemical characteristics of the soybean seed, including optimal seed protein concentration, seed sucrose concentration and seed weight [1–7], that are not of importance to commodity soybean breeding programs. As food processors require consistent seed composition to maintain production procedures, the development of environmentally stable, high yielding soybean cultivars with optimal value-added traits has become an important breeding objective.

Seed composition and yield component traits are affected by numerous genes and environmental factors [8–13]. Seed protein concentration shares a well-documented negative association with seed yield, which has hampered the development of competitive high-protein soybean cultivars [9, 14–23]. Additional value-added traits, such as high seed sucrose concentration and high seed weight, are also of interest to soy-food processors. Sucrose concentration is known to influence the palatability and texture of many soy-food products [24]. However, seed protein and sucrose concentrations share a significant inverse relationship [25]. This relationship can be detrimental for soy-foods, such as tofu, that require high concentrations of both protein and sucrose for optimal production [5]. The identification and use of quantitative trait loci (QTL) associated with elevated seed protein concentration and additional value-added traits could accelerate the development of competitive high-protein soybean cultivars for the North American food-grade market by accumulating desirable alleles into a common genetic background.

Numerous studies have sought to determine the genetic basis of seed protein accumulation in soybean. SoyBase has indexed 248 bi-parental QTL associated with seed protein concentration, which encompass the results of more than 35 independent studies [37]. These QTL are located on every soybean chromosome, although chromosomes 6, 15, 18 and 20 are particularly favoured [38]. A QTL-meta analysis conducted by Qi et al. [39] also identified 51 consensus QTL across numerous genetic backgrounds and growing environments, which were located on all linkage groups except Chromosome 16. Many factors, such as large confidence intervals, small additive effects, negative associations with other desirable traits, poor environmental stability and QTL-by-genetic background interaction effects, have limited the usefulness of these QTL in marker-assisted selection programs [40–44]. Numerous QTL have also been identified for other traits of interest, including 318 seed weight-related QTL identified in over 50 independent studies, and 188 seed yield-related QTL identified in 32 independent studies [37]. Sucrose concentration has received considerably less attention, with 37 sucrose-related QTL identified in 4 independent studies [37].

A global analysis of RNA-seq data revealed that Kunitz trypsin inhibitor 1, lectin family proteins, seed storage 2S albumin superfamily proteins, bZIP homologues and MYB-like transcription factors were associated with seed protein accumulation [39]. These transcripts were also associated with seed protein accumulation in previous studies [45–47]. Specific genes, such as *ABI3, ABI4* and *LEC1* have also been associated with seed protein accumulation [48, 49].

One method of detecting QTL that may be of use in improving polygenic traits is to utilize segregating populations derived from elite parents [46]. Previous studies aimed at detecting protein-related QTL have mostly used mapping populations derived from exotic germplasm or parental cultivars with large phenotypic differences for the desired traits [50]. Utilizing populations derived from elite lines may increase the chance of detecting novel QTL that were masked by common large-effect QTL in diverse populations. These QTL have a higher chance of being beneficial for the development of new high-protein soybean cultivars.

In the present study, two recombinant inbred line (RIL) populations derived from crosses involving three high-yielding soybean cultivars with high to moderately high-protein content were used to identify QTL associated with traits important for food-grade soybean. Significant genomic regions associated with seed protein concentration were examined for their relationship with seed sucrose concentrations, seed weight and yield. Identifying genomic regions that underlie multiple value-added traits would be beneficial for the simultaneous improvement of desirable traits in new food-grade soybean cultivars. To better understand the underlying mechanisms that regulate seed storage protein accumulation in soybeans, these regions were also screened for putative candidate genes.

## Results

### Phenotypic analyses of protein and other value-added food-grade traits

**Fig. 1:**
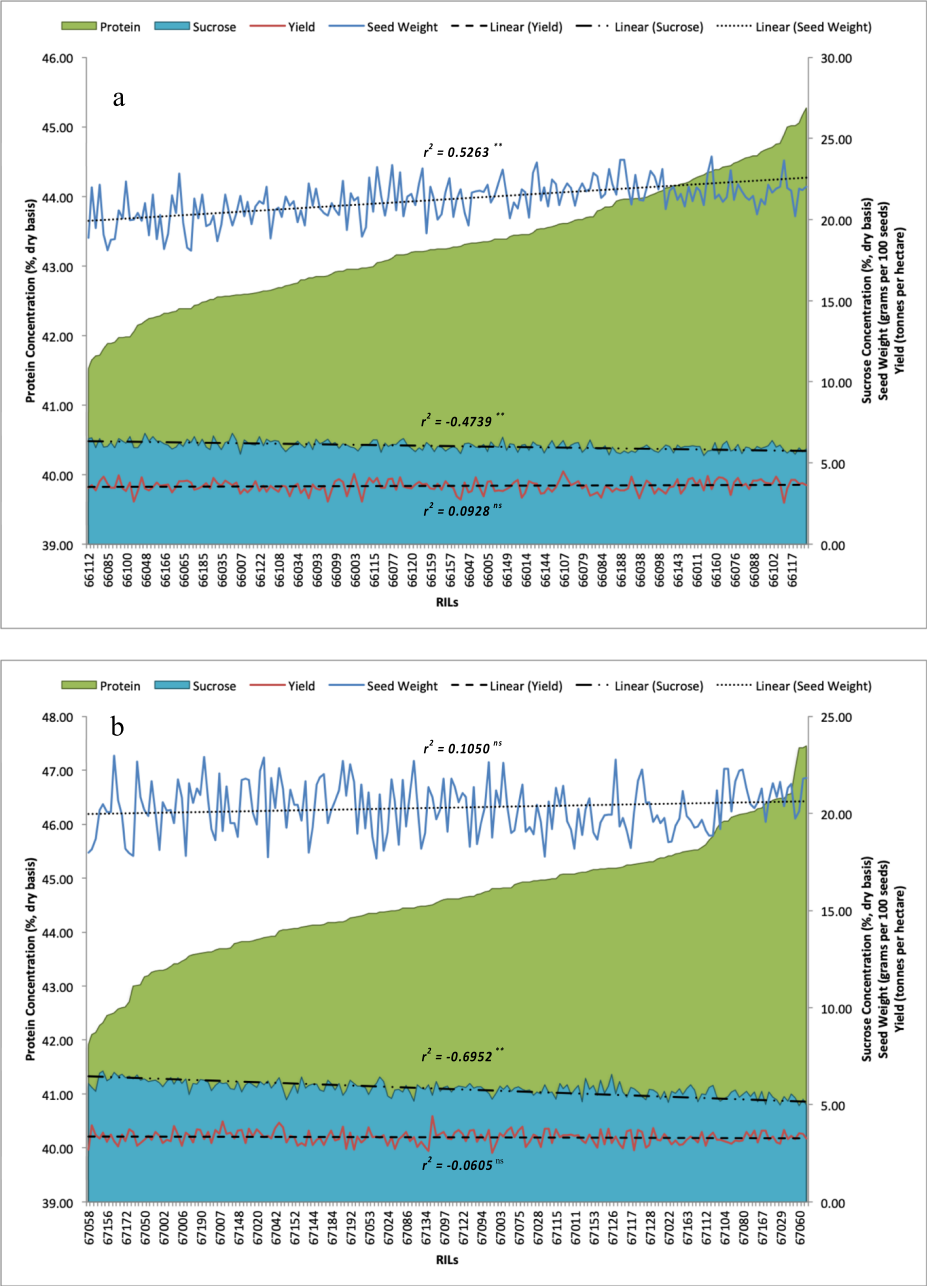
Relationship between average protein and sucrose concentrations (%, dry basis), seed weight (grams per 100 seeds) and seed yield (tonnes ha^− 1^) in RIL populations derived from (**a**) ‘AC X790P’ x ‘S18-R6’ and (**b**) ‘AC X790P’ x ‘S23-T5’ examined under combined Ontario environments in 2015 and 2016. Trendlines depict the linear regression between protein concentration and each trait. Pearson correlation coefficients are also noted (** denotes *p* < 0.05; ^ns^ denotes a non-significant relationship

The RIL populations were evaluated for seed weight, yield, protein and sucrose concentrations in multi-environment trials during the 2015 and 2016 field seasons (Fig. 1; Supplementary Table S1-S4). Seed protein and sucrose concentrations were measured using the high-throughput near-infrared reflectance (NIR) method, which is now a common way of measuring seed composition traits in soybean [51, 52]. Although the high-performance liquid chromatography (HPLC) is a more accurate way for measuring seed sucrose content, previous studies showed that NIR methods can also generate reliable and unbiased estimates for soybean seed sucrose concentration that are suitable for discriminating genotypes with different levels of sucrose and also for QTL studies [52]. In this study, contrasts were noted for seed protein concentration between the parental cultivars in both populations. In POPn_1, ‘AC X790P’ had an average protein concentration of 48.08% (± 0.19%, standard error) across the five testing environments, while ‘S18-R6’ had an average of 40.93% (± 0.19%). In POPn_2. ‘AC X790P’ had an average protein concentration of 48.24% (± 0.21%) across the five testing environments, while ‘S23-T5’ had an average of 42.60% (± 0.21%).

**Fig. 2:**
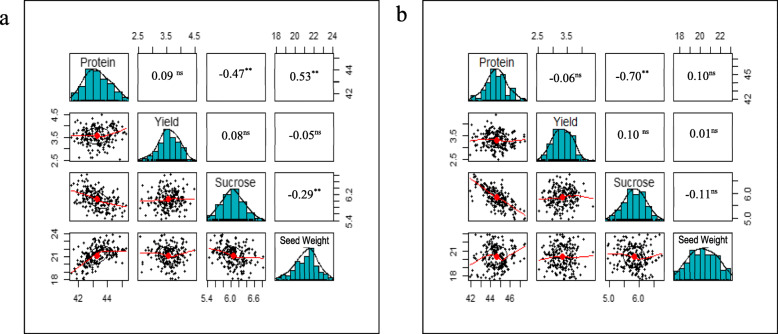
Distribution of LSMEANs and Pearson correlation coefficients among important seed quality traits in two RIL populations examined under combined Ontario environments in 2015 and 2016: (**a**) ‘AC X790P’ x ‘S18-R6’ and (**b**) ‘AC X790P’ x ‘S23-T5’

Differences in protein concentration between the RIL lines in each population were significant in the individual and combined multi-environment (Fig. 1; Supplementary Table S1). In POPn_1, seed protein concentration varied from 41.53 to 45.27%, with an average protein concentration of 43.31% (± 0.03%). In POPn_2, seed protein concentration varied from 41.93 to 47.46%, with an average protein concentration of 44.60% (± 0.03%) (Fig. 1; Supplementary Table S1). Transgressive segregation was observed in some individual environments but was not observed when the combined multi-environment data was considered (Supplementary Table S1). The normally distributed (Fig. 2) entry LSMEAN estimates indicate that protein concentration is controlled by multiple genes.

The parental cultivars also differed for seed yield, seed weight and seed sucrose concentration, and considerable variation was also noted within the combined multi-environment data for both populations (Fig. 1). In POPn_1, entry seed weight estimates (grams per 100 seeds) varied from 18.08 g to 23.88 g, with an average seed weight of 21.18 g (± 0.055 g). Seed yield also varied from 2.55 t ha^− 1^ to 4.49 t ha^− 1^, with an average seed yield of 3.57 t ha^− 1^ (± 0.025 t ha^− 1^) and seed sucrose concentration varied from 5.44 to 6.82%, with an average sucrose concentration of 6.06% (± 0.016%; Supplementary Table S2-S4). Similar variability was noted in POPn_2 (Fig. 1). Seed weight varied from 17.67 g to 22.95 g, with an average seed weight of 20.34 g (± 0.057 g). Seed yield varied from 2.52 t ha^− 1^ to 4.40 t ha^− 1^, with an average seed yield of 3.34 t ha^− 1^ (± 0.024 t ha^− 1^) and seed sucrose concentration varied from 4.95 to 6.75%, with an average sucrose concentration of 5.84% (± 0.014%). Transgressive segregation was noted for seed yield and seed sucrose concentration in both populations. While some RILs exhibited transgressive segregation in individual environments for seed weight, this was not observed when the combined multi-environment data was considered (Supplementary Table S2-S4).

Our previous study revealed significant differences (*p* < 0.01) in genotype, environment, and genotype x environment treatments for protein concentration and yield in these populations [53], which indicates the important role of genetic factors on the performance of these target traits. High heritability was noted for protein concentration and 100-seed weight (H^2^ = 0.93–0.95 and 0.87–0.89, respectively; Supplementary Table S5). Moderate heritability was observed for sucrose concentration (H^2^ = 0.70–0.81; Supplementary Table S5), and low heritability was observed for seed yield (H^2^ = 0.22–0.36) (Supplementary Table S5).

### Relationships between traits

Pearson’s correlation coefficients were used to determine the relationship between seed protein concentration and sucrose concentration, seed weight and yield in individual environments as well as combined multi-environment. Based on the combined multi-environment data, large, significant (α = 0.05) negative correlations were observed between seed protein and sucrose concentration in both populations (POPn_1: r = − 0.47; POPn_2: r = − 0.70; Fig. 2). In POPn_1, seed protein concentration and seed weight were positively correlated (POPn_1: r = 0.53), and seed weight and sucrose concentration were negatively correlated (POPn_1: r = − 0.29). Interestingly, no significant relationships were noted between seed protein concentration and seed yield in either population (POPn_1: r = 0.09; POPn_2: r = − 0.06) (Figs. 1 and 2). The linear relationship among the target agronomic and seed quality traits from individual environments are available in Supplementary Table S6.

### SNP mapping of the soybean genome

Linkage maps were constructed from polymorphic SNP markers in each population. In POPn_1, a linkage map was created using 807 polymorphic SNP markers, and divided into 39 linkage groups. A linkage map consisting of 1406 SNP markers on 40 linkage groups was created on POPn_2. All 20 chromosomes in the soybean genome were represented, with most chromosomes consisting of two or more linkage groups. The linkage maps were 2385 and 2690 cM in length for POPn_1 and POPn_2, respectively. The number of linkage groups was attributed to a lack of polymorphic markers between the parental genotypes distributed over large chromosomal regions, as elite Canadian soybean cultivars may share similar pedigrees.

### QTL associated with seed protein concentration

Using combined multi-environment data, 14 large-effect QTL were identified associated with seed protein concentration on Chromosomes 1, 2, 4, 5, 6, 8, 12, 13, 15 and 18. All the QTL were associated with protein in at least four individual environments. These 14 QTL explained between 10.4 and 21.9% of the observed phenotypic variation of seed protein concentration measured from combined multi-environment data (Table 1). Six of these QTL – *qPro_Gm01–2*, *qPro_Gm04–3*, *qPro_Gm06–1*, *qPro_Gm06–3*, *qPro_Gm12–3*, and *qPro-Gm12–4* – carried the beneficial alleles from ‘S18-R6’ or ‘S23-T5’, while the remaining eight QTL – *qPro_Gm02–3*, *qPro_Gm04–4*, *qPro-Gm05–2*, *qPro_Gm06–6*, *qPro-Gm08–2*, *qPro-Gm13–4*, *qPro_Gm15–3,* and *qPro_Gm18–3* – carried the favorable alleles from ‘AC X790P’. Positive protein-related QTL alleles in different genetic backgrounds suggests that it may be possible to stack favorable alleles to develop superior high-protein progeny.

**Table 1:** Major putative QTL (R^2^ > 10.0%) associated with soybean seed protein concentration identified by multiple QTL mapping (MQM) in the two RIL populations (‘AC X790P x S18-R6’ and ‘AC X790P x S23-T5’) evaluated in five environments (CHA15, CHA16, MER15, MER16 and PAL16)

QTL Name^**a**^	Chr.	POPn	Flanking Markers		Size (cM)	LOD^**b**^	A^**c**^	R^**2**^ (%)	Source	References^**d**^
*qPro_Gm01–2*	1	2	S01_42371693	S01_42555910	2.19	4.56	0.4578	10.4	S23-T5	–
*qPro_Gm02–3*	2	1	S02_40793724	S02_41072417	4.58	5.16	0.4115	10.4	AC X790P	VAL_SMA_; 1,2
*qPro_Gm04–3*	4	2	S04_44592458	S04_45008840	1.64	5.25	0.4931	11.0	S23-T5	2, 3, 11
*qPro_Gm04–4*	4	1	S04_48435528	S04_49024162	14.21	6.03	0.3570	13.7	AC X790P	–
***qPro_Gm05–2***	**5**	**1**	**S05_38330071**	**S05_38993543**	**12.31**	**6.80**	**0.4132**	**14.2**	**AC X790P**	**VAL**_**SMA**_
*qPro_Gm06–1*	6	1	S06_19074	S06_699413	1.68	10.19	0.4408	21.9	S18-R6	–
***qPro_Gm06–3***	**6**	**1**	**S06_9128442**	**S06_11029737**	**19.08**	**5.51**	**0.3339**	**12.6**	**S18-R6**	**VAL**_**SMA**_
*qPro_Gm06–6*	6	1	S06_30639643	S06_33589987	0.28	5.80	0.3046	13.2	AC X790P	2, 5, 6, 7
***qPro_Gm08–2***	**8**	**1**	**S08_43864875**	**S08_43896183**	**2.25**	**5.38**	**0.3936**	**12.3**	**AC X790P**	**VAL**_**SMA**_
*qPro_Gm12–3*	12	1	S12_924424	S12_1147989	11.46	6.45	0.4943	11.6	S18-R6	–
*qPro_Gm12–4*	12	1	S12_3518939	S12_3666689	7.64	6.63	0.4757	12.0	S18-R6	–
***qPro_Gm13–4***	**13**	**2**	**S13_28227783**	**S13_28254683**	**4.46**	**8.54**	**2.2804**	**11.6**	**AC X790P**	**VAL**_**SMA**_
*qPro_Gm15–3*	15	2	S15_10218629	S15_10877491	1.64	5.63	0.6925	11.5	AC X790P	VAL_SMA_; 4,8,9,10
*qPro_Gm18–4*	18	1	S18_52660341	S18_53019901	18.54	4.50	0.2713	10.4	AC X790P	VAL_SMA_; 2

^a^QTL for the same trait detected in all individual environments (CHA15, CHA16, MER15, MER16 and PAL16) and the combined environment (GMET) with the same or overlapping marker interval was designated as one QTL. QTL highlighted in bold are novel QTL and were validated in the other RIL population

^b^LOD thresholds were calculated through a permutation test with 1000 iterations and a Type I error rate of 0.001

^c^Additive effects calculated as the absolute value of half the subtraction of the mean of genotypes with the ‘S18-R6’ (‘POPn_1’) or ‘S23-T5’ (POPn_2) allele (negative effect) from the mean of genotypes with the ‘AC X790P’ allele (positive allele)

^d^Indicating that the QTL was confirmed in the other RIL population through multiple QTL mapping (VAL_MQM_), single marker analysis (VAL_SMA_), and/or has been reported previously in the reference(s): 1. [31] 2. [30] 3. [32] 4. [28] 5. [34] 6. [36] 7. [35] 8. [26] 9. [29] 10. [27].11. [33]

Of the 14 QTL identified in this study, nine QTL – *qPro_Gm01–2* (R2 = 10.4%), *qPro-Gm04–4* (R2 = 13.7%), *qPro-Gm05–2* (R2 = 14.2%), *qPro_Gm06–1* (R2 = 21.9%), *qPro_Gm06–3* (R2 = 12.6%), *qPro_Gm08–2* (R2 = 12.3%), *qPro-Gm12–3* (R2 = 11.6%), *qPro-Gm12–4* (R2 = 12%), and *qPro_Gm13–4* (R2 = 11.6%) – were previously unreported and so are considered as novel QTL (Table 1; 26]. Four of these novel QTL were detected in both mapping populations (Table 1). The rest of the QTL that were co-localized with previously reported protein-related QTL on SoyBase are listed in Table 1; Supplementary Table S7.

### QTL associated with additional value-added traits

**Fig. 3:**
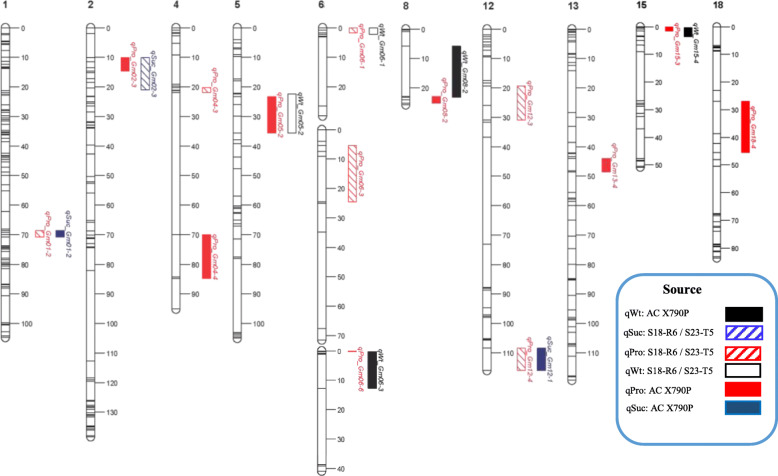
Graphical representation of putative QTL identified using multiple QTL mapping (MQM) algorithms for seed protein and sucrose concentrations, and seed weight in the two RIL populations: ‘AC X790P’ x ‘S18-R6’ and ‘AC X790P’ x ‘S23-T5’. Positive allele source is denoted by block pattern: ‘AC X790P’ is represented by a solid pattern, while ‘S18-R6’ and ‘S23-T5’ are represented by a striped pattern. Traits of interest are denoted by colour: seed protein concentration (red), seed sucrose concentration (navy) and seed weight (black)

Genomic regions harboring putative large-effect QTL associated with seed protein concentration were evaluated for their associations with seed yield, sucrose concentration, and seed weight using composite interval mapping analysis with the multiple QTL mapping (MQM) algorithm. (Table 2; Supplementary Table S8). Of the 14 protein-related QTL, eight QTL were co-localized with QTL associated with other traits. Three protein-related QTL – *qPro_Gm01–2*, *qPro_Gm02–3*, and *qPro_Gm12–4* – were co-localized with QTL associated with seed sucrose concentration (Table 2). The favorable alleles were inherited from opposing parental sources for each of these genomic regions, which supports the significant negative relationship observed between seed protein and sucrose concentration in this study. (Table 2; Fig. 3). The remaining five protein-related QTL were associated with seed weight, with positive associations noted for three of these regions (Table 2; Fig. 3). Favourable alleles were donated by each parental cultivar for all traits-of-interest. Protein-related QTL were not co-localized with significant regions for seed yield, consistent with the non-significant relationship between seed protein concentration and seed yield in both populations. SoyBase associated seven of our protein-related QTL with previously identified QTL for seed weight (nine QTL), seed oil concentration (five QTL) and seed yield (two QTL) (Supplementary Table S7 [37].

**Table 2:** Putative QTL for additional food-grade traits of interest (seed yield, seed weight and sucrose concentration) associated with major seed protein concentration QTL identified by multiple QTL mapping (MQM) in a RIL population derived from ‘AC X790P x S18-R6’ and ‘AC X790P x S23-T5’ examined under combined Ontario environments from 2015 and 2016

Protein QTL	QTL Name^**a**^	Chr.	POPn	Flanking Markers		Size (cM)	LOD^**b**^	A^**c**^	R^**2**^ (%)	Source	Relationship
qPro_Gm01–2	qSuc_Gm01–2	1	2	S01_42371693	S01_42555910	2.19	6.67	0.1472	14.5	AC X790P	Inverse
qPro_Gm02–3	qSuc_Gm02–3	2	2	S02_40716331	S02_42411031	11.17	5.46	0.1993	10.7	S23-T5	Inverse
qPro_Gm05–2	qWt_Gm5–2	5	2	S05_38273700	S05_38764985	1.94	3.98	1.2482	8.1	S23-T5	Inverse
qPro_Gm06–1	qWt_Gm6–1	6	1	S06_19074	S06_798961	2.24	4.46	0.3927	10.3	S18-R6	Positive
qPro_Gm06–6	qWt_Gm6–3	6	1	S06_30639643	S06_33589987	0.28	4.20	0.3754	9.4	AC X790P	Positive
qPro_Gm08–2	qWt_Gm8–2	8	1	S08_43325761	S08_43864912	17.39	4.29	0.5042	9.6	AC X790P	Positive
qPro_Gm12–4	qSuc_Gm12–1	12	1	S12_3518939	S12_3666689	7.64	5.49	0.1495	12.4	AC X790P	Inverse
qPro_Gm15–3	qWt_Gm15–4	15	2	S15_10731054	S15_11188445	3.33	2.78	0.8428	5.3	AC X790P	Positive

^a^QTL for the same trait detected in all individual environments (CHA15, CHA16, MER15, MER16 and PAL16) and the combined environment (GMET) with the same or overlapping marker interval was designated as one QTL

^b^LOD thresholds were calculated through a permutation test with 1000 iterations and a Type I error rate of 0.001

^c^Additive effects calculated as the absolute value of half the subtraction of the mean of genotypes with the ‘S18-R6’ (‘POPn_1’) or ‘S23-T5’ (POPn_2) allele (negative effect) from the mean of genotypes with the ‘AC X790P’ allele (positive allele)

### Candidate genes mining within protein QTL region

For further validation of the QTL identified as associated with seed protein concentration, a list of candidate genes was compiled using the Glyma 2.0 Assembly of Williams 82 on SoyBase (Wm82.a2.v1) according to their functional knowledge [37]. The number of genes in each QTL flanking region varied from four to seventy-four. In the flanking region corresponding to *qPro_Gm13–4* (spanning 26 kb), five genes were identified. These genes include Glyma.13G167800 and Glyma.13G167900, which are located 6 and 9 kb downstream of the SNP peak (28246299) and are annotated as a ribosomal protein and a ribosome biogenesis regulatory protein, respectively (Table 3). These genes have an indirect role in protein synthesis. Gene expression data provided by Severin et al. [54] noted that Glyma.13G167800 is expressed in the seed from 10 to 21 day after flowering (DAF). Glyma.13G167900 is also expressed in the seed albeit at a lower level compared to Glyma.13G167800. Two candidate genes, Glyma.06G004500 and Glyma.06G001800, underlying *qPro_Gm06–1* were identified. These genes, located in 74 kb upstream and 148 kb downstream of the QTL peak, respectively, encode transmembrane amino acid transporter proteins and ribosomal family proteins and (Table 3). Previous transcriptomic analyses noted increased expression of Glyma.06G004500 in the seed at 14 to 17, and 21 DAF [54].

**Table 3:** Major putative QTL (R^2^ > 10.0%) and candidate genes identified in confidence intervals of QTL associated with soybean seed protein concentration in the two RIL populations (‘AC X790P x S18-R6’ and ‘AC X790P x S23-T5’)

QTL Name^**a**^	Chr.	Flanking Markers	Candidate ID	Annotation	Type	Description	Position
*qPro_Gm02–3*	2	S02_40793724 - S02_41072417	Glyma.02 g220000	GO:0006412	GO-bp	60S Ribosomal protein L16p/L10e	40,794,106..40795066
			Glyma.02 g221500	GO:0006412	GO-bp	30S Ribosomal protein S2	40,921,208..40921756
*qPro_Gm04–4*	4	S04_48435528 - S04_49024162	Glyma.04 g212500	AT5G61750	AT	Cupin	48,435,108..48435965
			Glyma.04 g214500	GO:0006412	GO-bp	Ribosomal protein L17 family protein	
*qPro_Gm06–1*	6	S06_19074 - S06_699413	Glyma.06 g004500	GO:0015171	GO-mf	Transmembrane amino acid transporter protein	393,722..398436
			Glyma.06 g001800	GO:0006412	GO-bp	Ribosomal protein L3 family protein/Translation protein	171,462..172334
*qPro_Gm06–3*	6	S06_9128442 - S06_11029737	Glyma.06 g113700	GO:0006412	GO-bp	40S ribosomal protein S3a-like	9,225,152..9227191
			Glyma.06 g116400	PF01490	PFAM	Transmembrane amino acid transporter protein	9,472,699..9476835
			Glyma.06 g119700	GO:0006886	GO-bp	Intracellular protein transport	9,737,256..9743653
*qPro_Gm06–6*	6	S06_30639643 - S06_33589987	Glyma.06 g225600	GO:0006413	GO-bp	Translation initiation	31,131,372..31133932
			Glyma.06 g225700	GO:0006412	GO-bp	Translation initiation factor eIF-4F	31,209,402..31216702
*qPro_Gm13–4*	13	S13_28227783 - S13_28254683	Glyma.13 g167800	GO:0042254	GO-bp	Ribosome biogenesis	28,237,788..28239022
			Glyma.13 g167900	GO:0042254	GO-bp	Ribosome biogenesis regulatory protein	28,240,381..28243803
*qPro_Gm15–3*	15	S15_10218629 - S15_10877491	Glyma.15 g129800	GO:0006412	GO-bp	Ribosomal protein S27a/Ubiquitin family	10,430,457..10431571
			Glyma.15 g130000	GO:0006412	GO-bp	Structural constituent of ribosome	10,439,067..10440332
			Glyma.15 g134800	GO:0006412	GO-bp	Ribosomal protein L7/L12 C-terminal domain	10,831,146..10833232

^a^QTL for the same trait detected in all individual environments (CHA15, CHA16, MER15, MER16 and PAL16) and the combined environment (GMET) with the same or overlapping marker interval was designated as one QTL

Glyma.04G212500 and Glyma.04G214500 were identified under *qPro_Gm04–4* intervals*.* These genes are associated with the cupin superfamily and ribosomal protein family, respectively (Table 3). The cupin superfamily is involved in seed storage protein [55], while ribosomal protein family genes are associated with mRNA translation. In addition, candidate gene Glyma.04212500 are located exactly in the SNP peak position, which support the role of cupin associated with seed protein concentration. Glyma.06G113700, Glyma.06G116400, and Glyma.06G119700 were located in *qPro_Gm06–3* region (Table 3). Glyma.06G113700 encodes a potential structural constituent of 40S ribosomal protein. Glyma.06G116400 and Glyma.06G119700 were associated with a transmembrane amino acid transporter protein and an intracellular transport protein, respectively (Table 3).

Three candidate genes, Glyma.15G129800, Glyma.15G130000, and Glyma.15G134800, were identified from *qPro_Gm15–3* which are involved in structural constituents of the ribosome (Table 3). Moreover, Glyma.06G225600 and Glyma.06G225700, which were annotated as translation initiation factor proteins were identified under *qPro_Gm06–6* intervals (Table 3). Glyma.02G220000 and Glyma.02G221500, which contribute to the structural integrity of the ribosome and play a role in translation were located in *qPro_Gm02–3* region (Table 3). Based on previous transcriptomic analyses, Glyma.02G220000 is expressed in the seed 14 to 17, 21, 25, 28 and 35 DAF [54].

Candidate genes were also postulated for sucrose- and seed weight-related QTL that co-localized with protein-related regions. Four candidate genes were identified: Glyma.06G004400 and Glyma.06G007900, which were located under *qPro_Gm06–1* and *qWt_Gm06–1 region*, and Glyma.15G133600 and Glyma.15G133800 that were located under *qPro_Gm15–3* and *qWt_Gm15–4 region*. All four genes are involved in carbohydrate metabolism (GO:0005975) (Table 4).

**Table 4:** Major putative QTL (R^2^ > 10.0%) and candidate genes identified in confidence intervals of QTL associated with soybean seed protein concentration which co-located with seed weight or sucrose concentration in the two RIL populations (‘AC X790P x S18-R6’ and ‘AC X790P x S23-T5’)

Protein QTL	QTL Name	Chr.	Flanking Markers	Candidate ID	Annotation	Description	Position
*qPro_Gm06–1*	*qWt_Gm6–1*	6	S06_19074 - S06_798961	Glyma.06 g004400	GO:0005975	Carbohydrate metabolism	380,973..384365
				Glyma.06 g007900	GO:0005975	Carbohydrate metabolism	613,002..614426
*qPro_Gm15–3*	*qWt_Gm15–4*	15	S15_10731054 - S15_11188445	Glyma.15 g133600	GO:0005975	Carbohydrate metabolism	10,739,528..10743270
				Glyma.15 g133800	GO:0005975	Carbohydrate metabolism	10,754,838..10756823

## Discussion

Soy-based food manufacturers require specific physical and chemical characteristics of the soybean seed to maintain their production practices. For example, optimal tofu production requires high concentrations of both protein and sucrose in the soybean seed. However, protein and sucrose concentration have a negative relationship [38, 52, 56–58]. These significant negative relationships between seed protein concentration and other value-added traits have been major deterrents to the development of competitive food-grade soybean cultivars through conventional breeding methods [14–23, 59]. The identification of protein-related QTL that has no effect on sucrose or has a positive impact on other value-added traits would be of major benefit. The relationship between seed protein concentration, seed weight and yield in our study indicated that both current populations are desirable for the selection of optimal protein concentration with competitive yield and large seed size. On the other hand, negative relationship between seed protein and sucrose concentration indicated the selection for protein concentration may occur at the expense of seed sucrose concentration (and vice versa). These relationships could be attributed to tightly linked loci governing these traits separately, or to pleiotropic effects of specific loci [19].

Broad-sense heritability estimations in current study confirmed that a large proportion of the observed phenotypic variation for seed protein concentration, seed sucrose concentration, and seed weight are attributed to genotype. Therefore, phenotypic selection may be a successful tool to increase genetic gain for these traits. This is consistent with previous studies, in which moderate to high heritability estimates have been reported for seed protein concentration (H^2^ = 0.81–0.92; [16, 60], seed sucrose concentration (H^2^ = 0.46–0.86; [60, 61] and seed weight (H^2^ = 0.73–0.89; [60] across different genetic backgrounds and environments.

It is possible to ‘stack’ desirable QTL for multiple traits of interest using MAS, which allows breeders to screen early generation material for optimal trait combinations. This approach has been utilized breeding programs, especially for breeding disease resistance cultivars [62–64]. Maroof et al. [65] discussed the value of pyramiding race-specific soybean mosaic virus resistance genes using MAS, which involved the curation of specific genetic combinations for optimal multiple resistance. This approach increased the ability of the breeding program to select homozygous plants with multiple resistance, as the epistatic interactions among disease resistance genes made the phenotypic screening of disease reaction unreliable [65]. This strategy was also utilized by Jiang et al. [66], where the pyramiding of positive alleles from different parental sources was shown to increase seed protein filling rate and overall seed quality in soybean.

In this study, 14 large-effect QTL associated with seed protein concentration were identified, with the positive alleles derived from each of the parental sources. This may be attributed to the unique mapping populations utilized in this study. Previous QTL studies have used mapping populations that were derived from exotic germplasm or parental cultivars with large phenotypic differences for the desired trait-of-interest [50]. However, many modern elite soybean cultivars already possess high protein concentrations (approximately 40%, dry basis) and may be fixed for the large-effect QTL identified in diverse populations. In the current study, the utilization of moderate- and high-protein elite parental cultivars allowed for the identification of novel QTL that may have been masked in other populations [60, 67, 68] and also result in two or more linkage groups in most of chromosomes and the absence of major QTL regions associated with seed protein concentration, such as those on Chromosomes 15 and 20. The elimination of these regions may have also restricted the full scope of QTL interactions in these populations, and exaggerated the influence of the identified QTL on the traits-of-interest [67, 69, 70]. Additionally, many QTL mapping procedures have difficulty with the identification of small and intermediate effect QTL. These small and intermediate QTL are primarily associated with quantitative traits, such as seed protein concentration [71, 72]. The Beavis effect suggests that estimates of phenotypic variance may be greatly overestimated in smaller mapping populations (< 1000 progeny; 61), which may have further exaggerated the influence of the identified QTL in this study.

Recently, Hagely et al. [73] utilized direct molecular-assisted selection to improve the carbohydrate composition of soybean seeds. A natural variant of the raffinose synthase 3 gene (*rs3 snp5*) was associated with an ultra-low raffinose family oligosaccharide (UL RFO) carbohydrate profile, which improved the sucrose concentration and available metabolized energy of the soybean meal [74, 75]. The reduction in raffinose and stachyose was attributed to a specific genetic combination – *rs2 W331* + *rs3 snp5/rs3 snp 6* haplotype C – that results from a defect in the RS3 gene. Molecular marker assays were developed to detect these variants, which streamlined their introgression into elite soybean cultivars [73].

In an effort to further understand the underlying mechanisms of protein concentration in the soybean seed, candidate genes were identified from the flanking regions of our protein-related QTL and screened for their functional role in protein accumulation. In this study, 491 genes were identified and grouped using their biological process and functional annotation in SoyBase (www.soybase.org; [76]). Numerous putative candidate genes were identified (Table 4) through GO annotation: 16 genes were associated with protein translation processes (GO:0006412, GO:0015171, GO:0006413, GO:0042254, GO:0006886, AT6G61750, and PF01490), eight genes were associated with carbohydrate metabolism (GO:0005975), three genes were associated with lipid metabolism (GO:0006629), and the remainder were involved in signal transduction, transport, biosynthetic processes, nucleic acid metabolism, photosynthesis and numerous other functions. The significant relationships between protein, oil and sucrose [38, 52, 55, 57] support the role of genes associated with lipid and carbohydrate metabolism, which were also identified in the flanking region of these protein-related QTL.

Transcriptome analysis data provided by Severin et al., [54] showed Glyma.13G167800 (ribosome biogenesis), Glyma.13G167900 (ribosome biogenesis), Glyma.06G004500 (transmembrane amino acid transporter protein) and Glyma.02G220000 (60S ribosomal protein) are expressed in the seed, which supports their role in soybean seed protein accumulation. Glyma.04G212500 was associated with the cupin superfamily, which includes the 11S (glycine) and 7S (ß-conglycinin) seed storage proteins. 11S and 7S seed storage proteins account for ~ 70% of storage proteins within the soybean seed [54, 77]. Therefore, Glyma.04G212500 may have a strong association with seed protein accumulation in soybean. Zhang et al. [78] identified 13 candidate genes with putative roles in protein biosynthesis on Chromosome 15 and 20, with functional annotation of a structural constituent of ribosome, 60S ribosomal protein, amino acid transmembrane transport, and translation initiation factor 3. These annotations were also associated with seven candidate genes in our study, which strongly supports their role in protein accumulation in our populations. Zhang et al. [78] also conducted gene expression analyses of ribosomal, translation initiation factor 3 and amino acid transmembrane transport genes, which showed significant up-regulation of expression in the high-protein parent during the reproductive growth stage in the pod. This is consistent with their role in protein accumulation in soybean seeds [78]. Li et al. [79] also found a candidate gene in the flanking region of a protein QTL on chromosome 9, which was annotated as an amino acid transporter gene. In another study, the overexpression of one amino acid transporter gene in *Vicia narbonensis* and pea resulted in significant increases in seed protein concentration [80]. Further exploration of these candidate genes and their possible variants would further our understanding of protein accumulation pathways in the soybean seed and may lead to improved marker- or molecular-assisted breeding techniques for the improvement of soybean seed composition traits.

## Conclusion

In summary, nine of the protein-related QTL identified in this study were validated and may be suitable for marker assisted selection programs. Each provide vital information for the simultaneous improvement of multiple traits. Their value will be dictated by the objective of the individual breeding program. For example, *qPro_Gm06–1, qPro_Gm06–6, qPro_Gm08–2, and qPro_Gm15–3* were positively associated with seed weight QTL. These QTL may be unsuitable for a natto breeding program, which would favour smaller seed size. In this case, *qPro_Gm05–2* – a protein-related QTL inversely associated with seed weight – would be preferable. A curated panel of multiple-trait QTL may allow breeders to screen early-generation germplasm for the specific physical and chemical characteristics required by soy-food processors.

Future studies may look to consider the impact of protein biosynthesis, storage and metabolism on seed protein concentration in soybean, as suggested by the postulated candidate gene functions noted in this study, to foster a better understanding of protein accumulation pathways in the soybean seed. Breeders may also wish to dive deeper and explore the potential variants of these candidate genes, and their role in plant metabolism. The QTL presented in this study are offered as a tool for food-grade soybean breeding programs utilizing marker-assisted selection, and as a starting point for the discovery of variants in the protein biosynthesis pathway.

## Methods

### Mapping populations

Two populations of F_4_-derived recombinant inbred lines (RILs) were used to identify putative quantitative trait loci (QTL) for seed composition traits and yield. The first population (POPn_1) consisted of 190 RILs derived from a cross between ‘AC X790P’ and ‘S18-R6’. ‘AC X790P’ is a 2.2 relative maturity group (MG) cultivar developed by Agriculture and Agri-Food Canada in Harrow, Ontario, with a high, stable seed protein concentration (48.6%, dry weight basis; [49]). ‘S18-R6’ is a 1.8 MG commercial cultivar with a moderate seed protein concentration (40.4%), developed by Syngenta Canada, Inc. in Arva, Ontario [81].

The second population (POPn_2) was comprised of 193 RILs from a cross between ‘S23-T5’ and ‘AC X790P’. ‘S23-T5’ is a high-yielding 2.3 MG elite cultivar with moderate seed protein (41.3%) developed by Syngenta Seeds, Inc. in Owatonna, Minnesota [82]. Parental cultivars were considered high yielding when compared to the historical yield for southwestern Ontario [83]. Both RIL populations were developed at the University of Guelph, Ridgetown Campus.

### Experimental design

The RIL populations were grown in five environments across southwestern Ontario in 2015 and 2016: Chatham 2015 (CHA15), Merlin 2015 (MER15), Chatham 2016 (CHA16), Merlin 2016 (MER16) and Palmyra 2016 (PAL16). Field trials were planted using randomized complete block designs with two replications, in which the plot performance was adjusted for spatial variability through nearest neighbour analysis (NNA) using information from the immediate neighbouring plots in each of the five environments [53]. Plots consisted of five 4-m rows with 43-cm row spacing and were trimmed to 3.8-m in length following emergence. Plots were seeded at a rate of 69 seeds/m^2^ or 500 seeds per plot. Trials were maintained using standard tillage and cultural practices, and the three center rows of each plot were harvested for seed yield estimation and post-harvest evaluations.

### Phenotypic data collection

Seed protein and sucrose concentrations were determined for each harvested plot using a Perten DA 7250 SD near-infrared reflectance (NIR) analyzer (Perten Instruments Canada, Winnipeg, MB) using calibrations provided by Perten Instruments [84–87]. The calibration statistics for different seed composition traits, including seed protein and sucrose concentrations, are provided in Supplementary Table S9. Each NIR measurement is an average of three technical replications. Seed yield (tonnes ha^−1^at 13% moisture) and seed weight (grams per 100 seeds) were also recorded for each harvested plot.

### Statistical analyses

Statistical analyses were performed using SAS 9.4 (SAS Institute Inc., Cary, NC). An analysis of variance (ANOVA) was conducted and PROC MIXED was used to generate LSMEANS for each environment with ‘genotype’ as a fixed effect and ‘block’ as a random effect. PROC MIXED was also used to perform combined ANOVAs for seed weight, and protein and sucrose concentrations using the model:

$$ {Y}_{ij}=\mu +{\alpha}_i+{\beta}_j+{\alpha \beta}_{ij}+{\varepsilon}_{ij},j=1,\dots, n;i=1,\dots, k $$

where *Y*_*ij*_ represented the trait of interest (seed protein accumulation, seed sucrose accumulation, seed weight or seed yield), *α*_*i*_ represents the ‘genotype’ effect, *β*_*j*_ represents the ‘environment’ effect, *αβ*_*ij*_ represents the ‘genotype-by-environment’ effect and *ε*_*ij*_ represented the residual effect. ‘Genotype’, ‘environment’ and ‘genotype-by-environment’ were considered fixed effects and ‘block(environment)’ was considered a random effect. PROC CORR was used to examine the relationships between entry trait estimates.

### Genotypic data collection

Young trifoliate leaf tissue was collected from the first replicate block of each population at the Palmyra 2016 location. Leaf tissue for each RIL was sampled from multiple plants in each plot and stored in 2 mL screw cap tubes. The samples were freeze-dried for 72-h using a Savant ModulyoD Thermoquest (Savant Instruments, Holbrook, NY), and then stored at − 80 °C for future use. Genomic DNA was extracted from the freeze-dried tissue samples using a modified procedure from the Sigma GenElute™ DNA Extraction Kit (SIGMA®, Saint Louis, MO) methodology. DNA quality was verified using electrophoresis with 1% agarose gels, while quantity was verified using a Qubit® 2.0 fluorometer (Invitrogen, Carlsbad, CA)**.**

DNA samples (30 μl of 10 ng μl^− 1^ DNA) were transferred to Plate-forme D’analyses Génomiques at Université Laval (Laval, Quebec, Canada) for genotyping-by-sequencing (GBS), using the Fast-GBS pipeline with the *Gmax_275_v2* reference genome [88]. The Fast-GBS pipeline identified 24,738 high-quality single-nucleotide polymorphisms (SNPs). Heterozygous SNPs were considered missing data. SNPs with > 20% missing data or a minimum minor allele frequency less than 0.3 were discarded prior to imputation with Beagle [89].

### Linkage map construction and QTL mapping

JoinMap 5.0 software was used to construct genetic linkage maps for each population [90]. SNP markers with significant levels of segregation distortion that differed from the expected 1:1 ratio based on a chi-square test (α = 0.01) were removed from further analysis. Markers that segregated identically within the population were reduced to a single marker for linkage map construction. Markers were grouped into linkage groups within each chromosome using a minimum likelihood of odds (LOD) ≥ 3, and Kosambi’s mapping function was used to calculate genetic distances. Thereafter, the genetic position of these markers was anchored on physical position.

Composite interval mapping (CIM) was performed for the traits of interest using the multiple QTL mapping (MQM) algorithm in MapQTL® 6 [91]. The empirical LOD threshold values were calculated through a permutation test with 1000 iterations and a Type I error rate of 0.05. The automatic cofactor selection function was used to identify significant cofactors for MQM. Graphic representations of significant QTL were created using MapChart 2.32 [92].

Putative QTL regions associated with seed protein concentration were also screened for significant QTL associated with seed weight, seed yield and seed sucrose concentration. SoyBase was used to compare the putative QTL to published genomic regions related to seed protein concentration [38]. Putative QTL were also confirmed in the alternate population using single marker analysis (SMA) in SAS 9.4 (SAS Institute Inc., Cary, NC). PROC GLM was used to identify significant single marker effects (α < 0.0001) with LSMEAN estimates as the dependent variable and SNP marker as the independent variable. The SNP positions from genotype-by-sequencing were used to denote marker names in MQM and SMA.

### Candidate gene search

The flanking markers of each QTL were chosen based on the LOD values surrounding each peak marker. To ensure that the actual QTL was located within the range selected, the first marker below the LOD threshold on each side of the QTL peak was selected as the flanking marker. For each of the protein-related QTL, the regions between the flanking markers were used to identify candidate genes according to their function. A total of 491 genes were extracted from the flanking regions using the SoyBase Soybean Genetic Map. The functional annotation of each gene was identified from TAIR (www.arabidopsis.org/), GO (http://geneontology.org/), PFAM (http://pfam.xfam.org/), and PANTHER (http://www.pantherdb.org/) through SoyBase (https://soybase.org/). This functional knowledge used to reduce number of genes and identify putative candidate genes.

The Electronic Fluorescent Pictograph (eFP) browser for soybean (www.bar.utoronto.ca) was used to generate additional information about the candidate genes, such as tissue- and developmental-stage dependent expression (based on transcriptomic data from Severine et al. [54]). Pfam, a comprehensive collection of protein domains and families, and NCBI were used to obtain additional information about candidate genes.

## Supplementary information

**Additional file 1 : Supplementary Table S1.** Mean, standard error (α = 0.05), range, and parental means for soybean seed protein concentration (%, dry weight basis) in two RIL populations, ‘AC X790P’ x ‘S18-R6’ and ‘AC X790P’ x ‘S23-T5’, in five environments: Chatham 2015, Chatham 2016, Merlin 2015, Merlin 2016 and Palmyra 2016. **Supplementary Table S2.** Mean, standard error (α = 0.05), range, and parental means for soybean seed yield (tonnes ha^− 1^) in two RIL populations, ‘AC X790P’ x ‘S18-R6’ and ‘AC X790P’ x ‘S23-T5’, in five environments: Chatham 2015, Chatham 2016, Merlin 2015, Merlin 2016 and Palmyra 2016. **Supplementary Table S3.** Mean, standard error (α = 0.05), range, and parental means for soybean seed weight (100 seed weight in grams) in two RIL populations, ‘AC X790P’ x ‘S18-R6’ and ‘AC X790P’ x ‘S23-T5’, in five environments: Chatham 2015, Chatham 2016, Merlin 2015, Merlin 2016 and Palmyra 2016. **Supplementary Table S4.** Mean, standard error (α = 0.05), range, and parental means for soybean seed sucrose concentration (%, dry basis) in two RIL populations, ‘AC X790P’ x ‘S18-R6’ and ‘AC X790P’ x ‘S23-T5’, in five environments: Chatham 2015, Chatham 2016, Merlin 2015, Merlin 2016 and Palmyra 2016. **Supplementary Table S5.** Broad-sense heritability of protein concentration, sucrose concentration, seed weight and seed yield in two RIL populations evaluated in five environments (CHA15, CHA16, MER15, MER16 and PAL16). **Supplementary Table S6.** Pearson correlation coefficients for seed protein and sucrose concentrations, 100-seed weight, and seed yield in five environments (Chatham 2015, Chatham 2016, Merlin 2015, Merlin 2016, and Palmyra 2016) as well as the combined environment for the recombinant inbred line populations. **Supplementary Table S7.** Summary of major putative QTL (R^2^ > 10.0%) associated with soybean seed protein concentration, sucrose concentration and seed weight with potential use in marker-assisted selection, candidate genes, and co-localized QTL in the previous studies. **Supplementary Table S8.** Major putative QTL (R^2^ > 10.0%) associated with soybean seed sucrose concentration, yield and seed weight with potential use in marker-assisted selection, identified by multiple QTL mapping (MQM) in RIL populations examined under combined Ontario environments in 2015 and 2016. **Supplementary Table S9.** Whole-seed (dry basis) calibration values for oil, protein, Moisture and fatty acid components, as provided by Perten Instruments. The coefficient of determination for cross-validation (R^2^CV) explains the proportion of variance that can be predicted between reference chemistry and predicted values. The minimum and maximum values are the limits of the prediction range. The SECV is the standard error of cross validation, where samples are removed from the validation set and predicted, and the total error for the dataset is calculated. Factors shows the number of factors included in the calibration equation for a given trait. Samples shows the total number of samples used in the calibration. Calibration file dates for each trait are listed.

## Data Availability

The phenotypic and genotypic data that support the findings of this study are openly available in figshare at 10.6084/m9.figshare.13008467.

## Abbreviations

QTL: Quantitative trait loci
MAS: Marker-assisted selection
RIL: Recombinant inbred line
DAF: Day after flowering
POPn_1: First population
POPn_2: Second population
MG: Maturity group
CHA15: Chatham 2015
MER15: Merlin 2015
CHA16: Chatham 2016
MER16: Merlin 2016
PAL16: Palmyra 2016
NNA: Nearest neighbour analysis
NIR: Near-infrared reflectance
ANOVA: Analysis of variance
SNPs: Single-nucleotide polymorphisms
CIM: Composite interval mapping
MQM: Multiple QTL mapping
LOD: Likelihood of odds
SMA: Single marker analysis
eFP: electronic Fluorescent Pictograph
