# Supplementary material for: Genomic regions associated with important seed quality traits in food-grade soybeans
Source: BMC Plant Biol. 2020 Oct 23;20:485. doi: 10.1186/s12870-020-02681-0 (PMC7583236; doi:10.1186/s12870-020-02681-0)
Supplement: Supplementary file 1 — Additional file 1 : Supplementary Table S1. Mean, standard error (α = 0.05), range, and parental means for soybean seed protein concentration (%, dry weight basis) in two RIL populations, ‘AC X790P’ x ‘S18-R6’ and ‘AC X790P’ x ‘S23-T5’, in five environments: Chatham 2015, Chatham 2016, Merlin 2015, Merlin 2016 and Palmyra 2016. Supplementary Table S2. Mean, standard error (α = 0.05), range, and parental means for soybean seed yield (tonnes ha− 1) in two RIL populations, ‘AC X790P’ x ‘S18-R6’ and ‘AC X790P’ x ‘S23-T5’, in five environments: Chatham 2015, Chatham 2016, Merlin 2015, Merlin 2016 and Palmyra 2016. Supplementary Table S3. Mean, standard error (α = 0.05), range, and parental means for soybean seed weight (100 seed weight in grams) in two RIL populations, ‘AC X790P’ x ‘S18-R6’ and ‘AC X790P’ x ‘S23-T5’, in five environments: Chatham 2015, Chatham 2016, Merlin 2015, Merlin 2016 and Palmyra 2016. Supplementary Table S4. Mean, standard error (α = 0.05), range, and parental means for soybean seed sucrose concentration (%, dry basis) in two RIL populations, ‘AC X790P’ x ‘S18-R6’ and ‘AC X790P’ x ‘S23-T5’, in five environments: Chatham 2015, Chatham 2016, Merlin 2015, Merlin 2016 and Palmyra 2016. Supplementary Table S5. Broad-sense heritability of protein concentration, sucrose concentration, seed weight and seed yield in two RIL populations evaluated in five environments (CHA15, CHA16, MER15, MER16 and PAL16). Supplementary Table S6. Pearson correlation coefficients for seed protein and sucrose concentrations, 100-seed weight, and seed yield in five environments (Chatham 2015, Chatham 2016, Merlin 2015, Merlin 2016, and Palmyra 2016) as well as the combined environment for the recombinant inbred line populations. Supplementary Table S7. Summary of major putative QTL (R2 > 10.0%) associated with soybean seed protein concentration, sucrose concentration and seed weight with potential use in marker-assisted selection, candidate genes, and co-localized QTL in the p [file 12870_2020_2681_MOESM1_ESM.docx]

**Genomic Regions Associated with Important Seed Quality Traits in Food-grade Soybeans**

Rachel M.Whiting, Sepideh Torabi, Lewis Lukens, Milad Eskandari*

*Department of Plant Agriculture, University of Guelph, ON, Canada*

*Corresponding author Email: meskanda@uoguelph.ca

**Supplementary Table S1** Mean, standard error (α = 0.05), range, and parental means for soybean seed protein concentration (%, dry weight basis) in two RIL populations, ‘AC X790P’ x ‘S18-R6’ and ‘AC X790P’ x ‘S23-T5’, in five environments: Chatham 2015, Chatham 2016, Merlin 2015, Merlin 2016 and Palmyra 2016

| **POPn_1** | **Environment** | **Mean (Std. Error)** | **Range** | **AC X790P** | **S16-R6** |
| --- | --- | --- | --- | --- | --- |
|  | Chatham 2015 | 43.13 (0.058) | 39.42 – 46.51 | 48.16 | 39.89 |
|  | Chatham 2016 | 42.86 (0.051) | 40.44 – 45.62 | 48.01 | 41.58 |
|  | Merlin 2015 | 42.84 (0.052) | 40.46 – 45.72 | 48.33 | 40.66 |
|  | Merlin 2016 | 43.62 (0.051) | 40.77 – 45.95 | 47.84 | 41.31 |
|  | Palmyra 2016 | 44.08 (0.056) | 41.73 – 47.26 | 48.04 | 41.24 |
|  | **Combined** | **43.31 (0.026)** | **41.53 – 45.27** | **48.08** | **40.93** |
|  |  |  |  |  |  |
| **POPn_2** | **Environment** | **Mean (Std. Error)** | **Range** | **AC X790P** | **S23-T5** |
|  | Chatham 2015 | 44.63 (0.073) | 40.39 – 48.58 | 49.31 | 42.74 |
|  | Chatham 2016 | 43.59 (0.061) | 40.62 – 47.49 | 47.56 | 41.15 |
|  | Merlin 2015 | 44.55 (0.065) | 41.24 – 48.01 | 48.48 | 42.16 |
|  | Merlin 2016 | 44.88 (0.062) | 41.69 – 48.74 | 47.94 | 43.51 |
|  | Palmyra 2016 | 45.35 (0.063) | 41.48 – 48.74 | 47.92 | 43.44 |
|  | **Combined** | **44.60 (0.032)** | **41.93 – 47.46** | **48.24** | **42.60** |
|  |  |  |  |  |  |

**Supplementary Table S2** Mean, standard error (α = 0.05), range, and parental means for soybean seed yield (tonnes ha^-1^) in two RIL populations, ‘AC X790P’ x ‘S18-R6’ and ‘AC X790P’ x ‘S23-T5’, in five environments: Chatham 2015, Chatham 2016, Merlin 2015, Merlin 2016 and Palmyra 2016

| **POPn_1** | **Environment** | **Mean (Std. Error)** | **Range** | **AC X790P** | **S16-R6** |
| --- | --- | --- | --- | --- | --- |
|  | Chatham 2015 | 3.19 (0.067) | 0.45 – 5.73 | 2.92 | 2.16 |
|  | Chatham 2016 | 4.90 (0.026) | 3.64 – 6.45 | 4.42 | 5.42 |
|  | Merlin 2015 | 3.68 (0.049) | 0.80 – 5.09 | 3.36 | 3.05 |
|  | Merlin 2016 | 2.90 (0.029) | 1.78 – 5.09 | 2.17 | 3.14 |
|  | Palmyra 2016 | 3.18 (0.025) | 2.20 – 3.94 | 2.45 | 4.11 |
|  | **Combined** | **3.57 (0.025)** | **2.55 – 4.49** | **3.06** | **3.57** |
|  |  |  |  |  |  |
| **POPn_2** | **Environment** | **Mean (Std. Error)** | **Range** | **AC X790P** | **S23-T5** |
|  | Chatham 2015 | 3.43 (0.081) | 0.76 – 5.83 | 3.03 | 3.99 |
|  | Chatham 2016 | 4.32 (0.033) | 2.55 – 5.50 | 4.07 | 5.35 |
|  | Merlin 2015 | 2.74 (0.039) | 0.98 – 4.00 | 2.95 | 3.27 |
|  | Merlin 2016 | 2.94 (0.025) | 1.91 – 3.84 | 2.91 | 3.38 |
|  | Palmyra 2016 | 3.21 (0.026) | 1.82 – 4.14 | 3.37 | 3.89 |
|  | **Combined** | **3.34 (0.024)** | **2.52 – 4.40** | **3.27** | **3.98** |
|  |  |  |  |  |  |

**Supplementary Table S3** Mean, standard error (α = 0.05), range, and parental means for soybean seed weight (100 seed weight in grams) in two RIL populations, ‘AC X790P’ x ‘S18-R6’ and ‘AC X790P’ x ‘S23-T5’, in five environments: Chatham 2015, Chatham 2016, Merlin 2015, Merlin 2016 and Palmyra 2016

| **POPn_1** | **Environment** | **Mean (Std. Error)** | **Range** | **AC X790P** | **S16-R6** |
| --- | --- | --- | --- | --- | --- |
|  | Chatham 2015 | 20.37 (0.235) | 15.59 – 24.05 | 22.95 | 16.90 |
|  | Chatham 2016 | 23.57 (0.235) | 13.80 – 27.40 | 26.30 | 21.85 |
|  | Merlin 2015 | 18.99 (0.235) | 15.25 – 21.90 | 20.30 | 17.15 |
|  | Merlin 2016 | 22.56 (0.235) | 18.73 – 27.76 | 25.51 | 20.59 |
|  | Palmyra 2016 | 20.38 (0.235) | 13.80 – 23.45 | 21.00 | 19.65 |
|  | **Combined** | **21.18 (0.055)** | **18.08 – 23.88** | **23.21** | **19.23** |
|  |  |  |  |  |  |
| **POPn_2** | **Environment** | **Mean (Std. Error)** | **Range** | **AC X790P** | **S23-T5** |
|  | Chatham 2015 | 19.66 (0.211) | 16.95 – 24.15 | 22.40 | 17.10 |
|  | Chatham 2016 | 22.85 (0.210) | 13.45 – 26.95 | 28.93 | 19.83 |
|  | Merlin 2015 | 17.75 (0.212) | 14.23 – 22.05 | 21.65 | 14.50 |
|  | Merlin 2016 | 21.59 (0.210) | 17.97 – 25.26 | 25.42 | 19.23 |
|  | Palmyra 2016 | 19.57 (0.210) | 14.15 – 23.15 | 22.70 | 17.05 |
|  | **Combined** | **20.34 (0.057)** | **17.67 – 22.95** | **24.22** | **17.54** |
|  |  |  |  |  |  |

**Supplementary Table S4** Mean, standard error (α = 0.05), range, and parental means for soybean seed sucrose concentration (%, dry basis) in two RIL populations, ‘AC X790P’ x ‘S18-R6’ and ‘AC X790P’ x ‘S23-T5’, in five environments: Chatham 2015, Chatham 2016, Merlin 2015, Merlin 2016 and Palmyra 2016

| **POPn_1** | **Environment** | **Mean (Std. Error)** | **Range** | **AC X790P** | **S16-R6** |
| --- | --- | --- | --- | --- | --- |
|  | Chatham 2015 | 6.25 (0.097) | 5.23 – 7.34 | 5.53 | 6.24 |
|  | Chatham 2016 | 6.15 (0.097) | 4.69 – 7.47 | 5.53 | 6.58 |
|  | Merlin 2015 | 6.16 (0.097) | 5.14 – 7.50 | 5.18 | 6.14 |
|  | Merlin 2016 | 5.12 (0.097) | 4.32 – 6.03 | 4.76 | 5.38 |
|  | Palmyra 2016 | 6.61 (0.097) | 5.75 – 7.68 | 6.29 | 6.77 |
|  | **Combined** | **6.06 (0.016)** | **5.44 – 6.82** | **5.46** | **6.22** |
|  |  |  |  |  |  |
| **POPn_2** | **Environment** | **Mean (Std. Error)** | **Range** | **AC X790P** | **S23-T5** |
|  | Chatham 2015 | 6.27 (0.114) | 5.22 – 7.45 | 5.89 | 6.29 |
|  | Chatham 2016 | 5.71 (0.113) | 4.04 – 6.71 | 5.06 | 6.07 |
|  | Merlin 2015 | 5.61 (0.114) | 3.97 – 7.09 | 5.05 | 5.42 |
|  | Merlin 2016 | 5.52 (0.113) | 4.37 – 6.74 | 5.63 | 5.68 |
|  | Palmyra 2016 | 6.09 (0.113) | 4.18 – 7.59 | 5.79 | 5.84 |
|  | **Combined** | **5.84 (0.014)** | **4.95 – 6.75** | **5.48** | **5.86** |
|  |  |  |  |  |  |

**Supplementary Table S5** Broad-sense heritability of protein concentration, sucrose concentration, seed weight and seed yield in two RIL populations evaluated in five environments (CHA15, CHA16, MER15, MER16 and PAL16)

|  | **Protein** | **Yield** | **Seed Weight** | **Sucrose** |
| --- | --- | --- | --- | --- |
| **POPn_1** | 0.9275 | 0.3603 | 0.8648 | 0.7035 |
| **POPn_2** | 0.9501 | 0.2180 | 0.8924 | 0.8132 |

**Supplementary Table S6** Pearson correlation coefficients for seed protein and sucrose concentrations, 100-seed weight, and seed yield in five environments (Chatham 2015, Chatham 2016, Merlin 2015, Merlin 2016, and Palmyra 2016) as well as the combined environment for the recombinant inbred line populations.

| **POPn_1** | **CHA15** | Protein | Sucrose | Weight |  | **CHA16** | Protein | Sucrose | Weight |
| --- | --- | --- | --- | --- | --- | --- | --- | --- | --- |
|  | Yield | 0.33920** | 0.07712^ns^ | 0.45242** |  | Yield | -0.14197** | -0.19206** | 0.07585^ns^ |
|  | Weight | 0.47783** | -0.05822^ns^ |  |  | Weight | 0.24567** | -0.23595** |  |
|  | Sucrose | -0.34429** |  |  |  | Sucrose | -0.34774** |  |  |
|  |  |  |  |  |  |  |  |  |  |
|  | **MER15** | Protein | Sucrose | Weight |  | **MER16** | Protein | Sucrose | Weight |
|  | Yield | 0.28641** | 0.14804** | 0.33662** |  | Yield | -0.22836** | 0.16207** | 0.17084** |
|  | Weight | 0.45384** | -0.13800** |  |  | Weight | 0.41221** | -0.16303** |  |
|  | Sucrose | -0.23468** |  |  |  | Sucrose | -0.35883** |  |  |
|  |  |  |  |  |  |  |  |  |  |
|  | **PAL16** | Protein | Sucrose | Weight |  | **GMET** | Protein | Sucrose | Weight |
|  | Yield | -0.38017** | 0.09885* | 0.29201** |  | Yield | 0.09280^ns^ | 0.15572** | 0.36310** |
|  | Weight | 0.23137** | -0.16674** |  |  | Weight | 0.52630** | -0.30735** |  |
|  | Sucrose | -0.29171** |  |  |  | Sucrose | -0.47392** |  |  |
|  |  |  |  |  |  |  |  |  |  |
| **POPn_2** | **CHA15** | Protein | Sucrose | Weight |  | **CHA16** | Protein | Sucrose | Weight |
|  | Yield | 0.32387** | 0.15818** | 0.45408** |  | Yield | -0.15656** | 0.11900^ns^ | 0.15314** |
|  | Weight | 0.35170** | -0.08805^ns^ |  |  | Weight | 0.26598** | 0.03275^ns^ |  |
|  | Sucrose | -0.50189** |  |  |  | Sucrose | -0.50145** |  |  |
|  |  |  |  |  |  |  |  |  |  |
|  | **MER15** | Protein | Sucrose | Weight |  | **MER16** | Protein | Sucrose | Weight |
|  | Yield | 0.03432^ns^ | 0.00028^ns^ | 0.21973** |  | Yield | -0.16361** | 0.14708** | 0.20717** |
|  | Weight | 0.19175** | -0.20483** |  |  | Weight | 0.08484^ns^ | 0.07635^ns^ |  |
|  | Sucrose | -0.37639** |  |  |  | Sucrose | -0.57133** |  |  |
|  |  |  |  |  |  |  |  |  |  |
|  | **PAL16** | Protein | Sucrose | Weight |  | **GMET** | Protein | Sucrose | Weight |
|  | Yield | -0.21593** | 0.14572** | 0.26574** |  | Yield | -0.06051^ns^ | 0.12515** | 0.43606** |
|  | Weight | 0.06262^ns^ | 0.09917** |  |  | Weight | 0.10500^ns^ | -0.09426** |  |
|  | Sucrose | -0.34754** |  |  |  | Sucrose | -0.69523** |  |  |
|  |  |  |  |  |  |  |  |  |  |
| ^ns^ Non-significant  **Significant at α = 0.05  *Significant at α = 0.01 | | | | | | | | | |

**Supplementary Table S7** Summary of major putative QTL (R^2^ > 10.0%) associated with soybean seed protein concentration, sucrose concentration and seed weight with potential use in marker-assisted selection, candidate genes, and co-localized QTL in the previous studies

| **Protein QTL** | **Chr.** | **Flanking Markers** | | | **Candidate Genes** | **Co-localized QTL in the Previous Studies** | | | | |
| --- | --- | --- | --- | --- | --- | --- | --- | --- | --- | --- |
|  |  |  |  |  |  | **Protein** | **Seed Weight** | **Oil** | **Yield** | **References** |
| *qPro_Gm01-2* | 1 | S01_42371693 | - | S01_42555910 |  |  |  |  |  |  |
| *qPro_Gm02-3* | 2 | S02_40793724 | - | S02_41072417 | Glyma.02g220000  Glyma.02g221500 | Seed protein 40-5 |  |  |  | Qi et al. 2014 |
| *qPro_Gm04-3* | 4 | S04_44592458 | - | S04_45008840 |  | Seed protein 36-9  Seed protein 19-1  Seed protein 37-9 | Seed weight 47-3  Seed weight 36-15 |  |  | Mao et al.,2013  Stombaugh et al., 2004  Wang et al., 2014  Li et al., 2009  Han et al., 2012 |
| *qPro_Gm04-4* | 4 | S04_48435528 | - | S04_49024162 | Glyma.04g212500  Glyma.04g214500 |  |  |  |  |  |
| *qPro_Gm05-2* | 5 | S05_38330071 | - | S05_38993543 |  |  | Seed weight 34-9 |  |  | Han et al., 2012 |
| *qPro_Gm06-1* | 6 | S06_19074 | - | S06_699413 | Glyma.06g004500  Glyma.06g001800 |  |  |  |  |  |
| *qPro_Gm06-3* | 6 | S06_9128442 | - | S06_11029737 | Glyma.06g113700  Glyma.06g116400  Glyma.06g119700 |  |  |  |  |  |
| *qPro_Gm06-6* | 6 | S06_30639643 | - | S06_33589987 | Glyma.06g225600  Glyma.06g225700 | Seed protein 28-1  Seed protein 29-1  Seed protein 35-2  Seed protein 36-7  Seed protein 36-8 | Seed weight 19-1 |  |  | Liang et al.,2010  Palomeque et al.,2009  Rossie et al., 2013  Mao et al., 2013  Funatsuki et al., 2005 |
| *qPro_Gm08-2* | 8 | S08_43864875 | - | S08_43896183 |  |  |  |  |  |  |
| *qPro_Gm12-3* | 12 | S12_924424 | - | S12_1147989 |  |  | Seed weight 16-3  Seed weight 43-3 | Seed oil 44-2 |  | Funatsuki et al., 2005  Kuroda et al., 2013  Leit et al., 2016 |
| *qPro_Gm12-4* | 12 | S12_3518939 | - | S12_3666689 |  |  | Seed weight 43-3 | Seed oil 44-2 |  | Kuroda et al., 2013 |
| *qPro_Gm13-4* | 13 | S13_282277833 | - | S13_2825468 | Glyma.13g167800  Glyma.13g167900 |  |  |  |  |  |
| *qPro_Gm15-3* | 15 | S15_10218629 | - | S15_10877491 | Glyma.15g129800  Glyma.15g130000  Glyma.15g134800 | Seed protein 39-2  Seed protein 3-6  cqSeed protein-001  Seed protein 5-1 |  | Seed oil 24-24  mqSeed oil-013  Seed oil 27-2 |  | Warrington et al., 2015  Brummer et al., 1997  Fasoula et al., 2004  Lee et al., 2004  Qi et al., 2011  Reinprecht et al., 2006 |
| *qPro_Gm18-4* | 18 | S18_52660341 | - | S18_53019901 |  | Seed protein 36-25 | Seed weight 6-6  Seed weight 6-8 |  | Seed yield 27-3  Seed yield 30-3 | Mao et al.,2013  Yao et al., 2015  Kim et al., 2012 |

**Supplementary Table S8** Major putative QTL (R^2^ > 10.0%) associated with soybean seed sucrose concentration, yield and seed weight with potential use in marker-assisted selection, identified by multiple QTL mapping (MQM) in RIL populations examined under combined Ontario environments in 2015 and 2016

| **Trait** | **QTL Name^z^** | **Chr.** | **POPn** | **Flanking Markers** | | | **Size (cM)** | **LOD^y^** | **a^x^** | **R^2^ (%)** | **Source** |
| --- | --- | --- | --- | --- | --- | --- | --- | --- | --- | --- | --- |
| *Sucrose* | *qSuc_Gm01-1* | 1 | 1 | S01_150451 | - | S01_293924 | 2.30 | 5.70 | 0.1022 | 13.0 | S18-R6 |
|  | *qSuc_Gm01-2* | 1 | 2 | S01_42371693 | - | S01_42555910 | 2.19 | 6.67 | 0.1472 | 14.5 | AC X790P |
|  | *qSuc_Gm02-3* | 2 | 2 | S02_40716331 | - | S02_42411031 | 11.17 | 5.46 | 0.1993 | 10.7 | S23-T5 |
|  | *qSuc_Gm03-2* | 3 | 2 | S03_44917381 | - | S03_45395338 | 8.73 | 5.20 | 0.1255 | 11.7 | S23-T5 |
|  | *qSuc_Gm09-2* | 9 | 1 | S09_49279854 | - | S09_49546888 | 10.70 | 5.16 | 0.1700 | 11.9 | AC X790P |
|  | *qSuc_Gm12-1* | 12 | 1 | S12_3518939 | - | S12_3666689 | 7.64 | 5.49 | 0.1495 | 12.4 | AC X790P |
|  | *qSuc_Gm13-6* | 13 | 2 | S13_31531708 | - | S13_31639456 | 4.73 | 6.36 | 0.6121 | 11.2 | S23-T5 |
|  | *qSuc_Gm14-2* | 14 | 2 | S14_1086390 | - | S14_1282950 | 6.75 | 4.95 | 0.3898 | 10.2 | AC X790P |
|  | *qSuc_Gm16-1* | 16 | 2 | S16_37420716 | - | S16_37753573 | 1.64 | 6.69 | 0.1398 | 14.8 | S23-T5 |
|  | *qSuc_Gm18-1* | 18 | 1 | S18_49993979 | - | S18_50225033 | 9.60 | 4.59 | 0.1866 | 10.2 | S18-R6 |
|  |  |  |  |  |  |  |  |  |  |  |  |
| *Seed Yield* | *qYld_Gm15-4* | 15 | 2 | S15_24465408 | - | S15_25479719 | 2.22 | 5.98 | 1.6936 | 10.7 | S23-T5 |
|  | *qYld_Gm20-4* | 20 | 2 | S20_44438965 | - | S20_44771176 | 1.94 | 6.45 | 0.9447 | 12.5 | AC X790P |
|  |  |  |  |  |  |  |  |  |  |  |  |
| *Seed Weight* | *qWt_Gm02-1* | 2 | 2 | S02_1883298 | - | S02_2417746 | 14.93 | 4.85 | 0.9392 | 10.6 | S23-T5 |
|  | *qWt_Gm03-1* | 3 | 2 | S03_5818458 | - | S03_19256703 | 10.58 | 6.05 | 0.4838 | 13.5 | S23-T5 |
|  | *qWt_Gm04-1* | 4 | 1 | S04_9555350 | - | S04_9936875 | 2.54 | 5.04 | 0.4104 | 11.6 | S18-R6 |
|  | *qWt_Gm04-2* | 4 | 1 | S04_15438084 | - | S04_15954764 | 2.83 | 4.89 | 0.4088 | 11.3 | AC X790P |
|  | *qWt_Gm05-1* | 5 | 1 | S05_4293377 | - | S05_4392756 | 2.34 | 5.88 | 0.4526 | 13.4 | S18-R6 |
|  | *qWt_Gm06-1* | 6 | 1 | S06_19074 | - | S06_798961 | 2.24 | 4.46 | 0.3927 | 10.3 | S18-R6 |
|  | *qWt_Gm06-2* | 6 | **1** | S06_7995062 | - | S06_9128442 | 5.38 | 5.98 | 0.4536 | 13.1 | S18-R6 |
|  | *qWt_Gm07-2* | 7 | 2 | S07_2438965 | - | S07_2788084 | 3.96 | 4.89 | 0.5651 | 10.1 | AC X790P |
|  | *qWt_Gm08-1* | 8 | 1 | S08_42089038 | - | S08_43325761 | 5.83 | 5.59 | 0.5780 | 12.7 | S18-R6 |
|  | *qWt_Gm11-1* | 11 | **1** | S11_30385803 | - | S11_30425212 | 1.12 | 5.59 | 0.4344 | 12.8 | AC X790P |
|  | *qWt_Gm12-2* | 12 | 1 | S12_3358348 | - | S12_3518939 | 2.94 | 8.09 | 0.7896 | 15.8 | AC X790P |
|  | *qWt_Gm13-1* | 13 | 1 | S13_26716128 | - | S13_27766926 | 10.80 | 5.71 | 0.6586 | 12.9 | S18-R6 |
|  | *qWt_Gm14-4* | 14 | 2 | S14_3738849 | - | S14_4112800 | 10.60 | 5.03 | 1.1426 | 10.6 | AC X790P |
|  | *qWt_Gm14-6* | 14 | 1 | S14_6573275 | - | S14_6976251 | 10.34 | 7.90 | 0.6402 | 15.8 | S18-R6 |
|  | *qWt_Gm15-1* | 15 | 2 | S15_1248951 | - | S15_1528828 | 1.09 | 6.31 | 0.4874 | 14.0 | S23-T5 |
|  | *qWt_Gm15-2* | 15 | 1 | S15_6427154 | - | S15_6747765 | 4.75 | 4.55 | 0.5919 | 10.3 | S18-R6 |
|  | *qWt_Gm15-3* | 15 | 1 | S15_7552589 | - | S15_7756662 | 2.25 | 4.85 | 0.6126 | 11.1 | AC X790P |
|  | *qWt_Gm15-6* | 15 | **1** | S15_36957405 | - | S15_37104746 | 1.68 | 5.37 | 0.4245 | 12.3 | AC X790P |
|  | *qWt_Gm16-2* | 16 | 2 | S16_36255272 | - | S16_36735586 | 4.03 | 5.07 | 1.1719 | 11.3 | S23-T5 |
|  | *qWt_Gm18-1* | 18 | 1 | S18_2129860 | - | S18_2460283 | 6.39 | 5.11 | 0.4214 | 11.8 | S18-R6 |
|  | *qWt_Gm18-2* | 18 | 2 | S18_5212723 | - | S18_5214289 | 0.55 | 6.92 | 0.6039 | 15.3 | S23-T5 |
|  | *qWt_Gm19-1* | 19 | 1 | S19_49218060 | - | S19_49654021 | 13.70 | 5.42 | 0.7695 | 12.4 | AC X790P |
|  |  |  |  |  |  |  |  |  |  |  |  |
| ^z^QTL for the same trait detected in all individual environments (CHA15, CHA16, MER15, MER16 and PAL16) and the combined environment (GMET) with the same or overlapping marker interval was designated as one QTL. QTL highlighted in bold are novel QTL and were validated in the other RIL population.  ^y^LOD thresholds were calculated through a permutation test with 1,000 iterations and a Type I error rate of 0.001.  ^x^Additive effects calculated as the absolute value of half the subtraction of the mean of genotypes with the ‘S18-R6’ (‘POPn_1’) or ‘S23-T5’ (POPn_2) allele (negative effect) from the mean of genotypes with the ‘AC X790P’ allele (positive allele). | | | | | | | | | | | |

**Supplementary Table S9** Whole-seed (dry basis) calibration values for oil, protein, Moisture and fatty acid components, as provided by Perten Instruments. The coefficient of determination for cross-validation (R^2^CV) explains the proportion of variance that can be predicted between reference chemistry and predicted values. The minimum and maximum values are the limits of the prediction range. The SECV is the standard error of cross validation, where samples are removed from the validation set and predicted, and the total error for the dataset is calculated. Factors shows the number of factors included in the calibration equation for a given trait. Samples shows the total number of samples used in the calibration. Calibration file dates for each trait are listed.

| **Trait (%)** | **R^2^CV** | **Minimum** | **Maximum** | **SECV** | **Factors** | **Samples** | **Calibration File** |
| --- | --- | --- | --- | --- | --- | --- | --- |
| **Oil** | 0.83 | 13.6 | 26.5 | 0.8 | 19 | 3381 | 20170608 |
| **Protein** | 0.89 | 33.4 | 53.6 | 0.8 | 18 | 3607 | 20170608 |
| **Moisture** | 0.27 | 4.0 | 15.7 | 2.9 | 19 | 3531 | 20160609 |
| **Palmitic Acid** | 0.04 | 4.4 | 18.9 | 5.0 | 19 | 3490 | 20160609 |
| **Stearic Acid** | 0.60 | 2.4 | 6.9 | 0.5 | 19 | 3524 | 20160609 |
| **Oleic Acid** | 0.88 | 13.8 | 89.6 | 4.3 | 19 | 3423 | 20160609 |
| **Linoleic Acid** | 0.77 | 3.6 | 66.7 | 5.3 | 19 | 3434 | 20160609 |
| **Linolenic Acid** | 0.50 | 0.8 | 14.1 | 1.8 | 19 | 3514 | 20160609 |
| **Sucrose** | 0.33 | 1.2 | 10.3 | 1.2 | 20 | 1333 | 20160609 |
